# Defibrotide for the Treatment of Veno‐Occlusive Disease/Sinusoidal Obstruction Syndrome in Paediatric Patients Who Did Not Receive Haematopoietic Stem Cell Transplantation: Case Reports of Patients From a German Academic Hospital

**DOI:** 10.1002/jha2.1094

**Published:** 2025-01-23

**Authors:** Katharina Kleinschmidt, Anja Troeger, Jürgen Föll, Tarek Hanafee‐Alali, Marcus Jakob, Sonja Kramer, Silke Kietz, Selim Corbacioglu

**Affiliations:** ^1^ Department for Paediatric Haematology Oncology and Stem Cell Transplantation University Hospital Regensburg Regensburg Germany

**Keywords:** acute lymphoblastic leukaemia, chemotherapy, children, defibrotide, pEBMT diagnostic criteria for VOD/SOS, veno‐occlusive disease/sinusoidal obstruction syndrome

## Abstract

**Introduction:**

Veno‐occlusive disease/sinusoidal obstruction syndrome (VOD/SOS) is a potentially life‐threatening condition characterised by obstruction of the small veins of the liver. Although typically associated with haematopoietic stem cell transplantation, VOD/SOS may also occur following intensive multimodal chemotherapy regimens. In children, symptoms of VOD/SOS are refractory thrombocytopaenia, weight gain, hepatomegaly, ascites and fluid retention, hyperbilirubinaemia and sometimes right upper quadrant pain.

**Methods:**

Here, we present a case series of six paediatric patients with acute lymphoblastic leukaemia who developed severe VOD/SOS while receiving standard AIEOP‐BFM ALL protocol treatment.

**Results/Conclusions:**

All patients responded promptly to defibrotide treatment and exhibited favourable clinical outcomes.

**Trial Registration:**

The authors have confirmed clinical trial registration is not needed for this submission

## 1 Introduction

Veno‐occlusive disease/sinusoidal obstruction syndrome (VOD/SOS) is a potentially life‐threatening condition characterised by obstruction of the small veins of the liver [1, 2]. VOD/SOS is a frequent and often fatal complication of haematopoietic stem cell transplantation (HSCT), particularly in children [3]. It is also rarely reported to occur in patients receiving first‐line chemotherapy for diseases such as leukaemia, nephroblastoma or central nervous system tumours [4, 5, 6, 7, 8, 9]. The use of high‐intensity chemotherapy regimens, ozogamicin‐conjugated antibodies and/or total body irradiation are associated with increased risk of VOD/SOS, with the highest risk in infants [10]. A single‐arm, open‐label prospective study in 1137 patients with VOD/SOS (T‐IND study) recorded a 12% incidence of patients following non‐HSCT associated chemotherapy [4].

Clinical presentation of VOD/SOS includes refractory thrombocytopaenia (RT) as the earliest finding, weight gain, hepatomegaly, ascites and fluid retention, hyperbilirubinaemia (often as a late finding) and right upper quadrant pain [2]. The paediatric EBMT (pEBMT) criteria reflect particularities of VOD/SOS in children, allowing early diagnosis in this age group (Table 1) [2]. Given the main prevalence of VOD/SOS in transplant patients [3], awareness of this complication in the non‐transplant setting is low [6].

**TABLE 1:** pEBMT diagnostic criteria for VOD/SOS [2].

No time limitation for onset of SOS^a^Presence of ≥ 2 of the following^b^: Unexplained consumptive and transfusion‐refractory thrombocytopaenia Unexplained weight gain on 3 consecutive days despite the use of diuretics or a weight gain > 5% above baseline value^c^ Hepatomegaly (ideally confirmed by imaging) above baseline value^c^ Ascites (ideally confirmed by imaging) above baseline value^c^ Rising bilirubin from a baseline value^c^ on 3 consecutive days or ≥ 2 mg/dL within 72 h

Abbreviations: HSCT, haematopoietic stem cell transplantation; pEBMT, EBMT paediatric; SOS, sinusoidal obstruction syndrome; VOD, veno‐occlusive disease.

^a^ Up to 20% of paediatric patients present VOD/SOS > 30 days after HSCT.

^b^ With the exclusion of other potential differential diagnoses.

^c^ Baseline values should be determined immediately before HSCT.

Defibrotide (Defitelio) is approved in the European Union for treatment of severe hepatic VOD/SOS post‐HSCT in patients aged > 1 month [11] and in the United States for treatment of VOD/SOS in patients with renal or pulmonary dysfunction post‐HSCT [12]. Defibrotide is effective for prevention of VOD/SOS in children and treatment of VOD/SOS in children/adults for both post‐HSCT and non‐HSCT settings [4, 8, 13–15]. The T‐IND study demonstrated a survival rate of 59% with an acceptable safety profile [15]. In 82 non‐HSCT patients with VOD/SOS within 30 days post‐chemotherapy, the survival rate at 70 days post‐defibrotide was 74% [4]. In the AIEOP‐BFM ALL 2000 trial, 12/13 patients with acute lymphoblastic leukaemia (ALL) who developed VOD/SOS post‐thioguanine received defibrotide, and all recovered [7]. In addition, a case report of two paediatric patients diagnosed with non‐HSCT VOD/SOS post‐thioguanine for ALL reported complete resolution with defibrotide [8].

To emphasise awareness of non‐HSCT VOD/SOS, we present a case series of six children with ALL who developed VOD/SOS on standard AIEOP‐BFM ALL protocol treatment.

## 2 Methods and Case Studies

We describe the course of patients with VOD/SOS identified retrospectively by single‐centre data review, defibrotide use and clinical outcomes. During the time period analysed (February 2019 to April 2022), a total of 45 patients were treated on standard AIEOP‐BFM ALL protocols. Patient characteristics and details relating to the course of VOD/SOS and defibrotide treatment are detailed in Table 2. All patients were diagnosed with severe VOD/SOS according to pEBMT criteria (see Table 1 for the full pEBMT diagnostic criteria for VOD/SOS) and treated with defibrotide 25 mg/kg of body weight per day. No adverse events were reported during defibrotide treatment for any of these patients.

**TABLE 2:** Summary of patient characteristics and details relating to VOD/SOS diagnosis and defibrotide treatment.

	Patient 1	Patient 2	Patient 3	Patient 4	Patient 5	Patient 6
**Patient characteristics**						
Age	16 years	12 years	6 years	11 years	3 years	22 months
Sex	Male	Female	Female	Female	Female	Male
Diagnosis	T‐ALL	c‐ALL	Pre‐B‐ALL	Pre‐B‐ALL	c‐ALL	Pro‐B‐ALL
Treatment protocol	AIEOP‐BFM ALL 2017	AIEOP‐BFM ALL 2017	AIEOP‐BFM ALL 2017	AIEOP‐BFM ALL 2017	AIEOP‐BFM ALL 2009	AIEOP‐BFM ALL 2017
Treatment prior to VOD/SOS diagnosis	Protocol III (with thioguanine)	Consolidation A (with 6‐mercaptopurine)	Protocol IIIB (with thioguanine)	Protocol IA (with daunorubicin and vincristine)	Protocol IIB (with thioguanine)	Protocol IIB (with thioguanine)
**VOD/SOS diagnosis**						
Symptoms at onset of VOD/SOS: pEBMT diagnostic criteria						
Thrombocytopaenia	X^a^		X^a^	X	X^a^	X^a^
Weight gain					X^a^	X
Hepatomegaly		X	X	X	X	X
Ascites	X	X	X		X	X
Hyperbilirubinaemia	X	X^a^		X^a^		X
Other symptoms at onset of VOD/SOS						
Anaemia	X	X	X	X	X	X
Fever	X		X	X	X	
Coagulopathy	X	X		X	X	X
Increased abdominal girth	X		X		X	
Increased transaminases	X		X	X		X
Hepatic tenderness		X		X		
Pleural effusion	X		X			X
Decreased hepatic perfusion		X		X		
Reversal of portal venous flow	X		X		X	X
Compromised renal function			X			
VOD/SOS severity score	4	4	4	4	4	4
Symptoms leading to severity score						
ALT and AST levels > 5 times normal values	X			X		X
Hyperbilirubinaemia (levels of ≥ 2 mg/dL)	X	X		X	X	X
Ascites with need for paracentesis			X			X
Bilirubin levels doubling within 48 h	X			X	X	
Impaired coagulation with need for replacement of coagulation factors	X	X		X	X	X
Time to VOD/SOS diagnosis/start of defibrotide treatment (days)^b^	1	3	2	1	2	2
**Treatment of VOD/SOS**						
Defibrotide treatment duration (days)	14	11	14	8	18	17
Treatment outcome	CR	CR	CR	CR^c^	CR	CR
Time to discharge (days)	16	13	17	11	19	18

Abbreviations: ALT, alanine aminotransferase; AST, aspartate aminotransferase; c‐ALL, common B‐cell acute lymphocytic leukaemia; CR, complete response; pEBMT, EBMT paediatric; pre‐B‐ALL, pre‐B cell acute lymphoblastic leukaemia; pro‐B‐ALL, pro‐B cell acute lymphoblastic leukaemia; SOS, sinusoidal obstruction syndrome; T‐ALL, T‐cell acute lymphoblastic leukaemia; VOD, veno‐occlusive disease.

^a^ Earliest diagnostic criterion.

^b^ Based on time from presentation of the earliest diagnostic criterion to confirmed diagnosis and start of defibrotide treatment.

^c^ This patient later died after achieving CR.

### 2.1 Patient 1 (Male, 16 Years)

Patient 1 received cyclophosphamide, thioguanine and cytarabine based on AIEOP‐BFM ALL 2017 treatment protocol IIIB. Three days after the end of treatment, the patient presented initially with RT (the earliest symptom), anaemia, fever and coagulopathy; the next day the patient developed increased abdominal girth, ascites, hyperbilirubinaemia (up to 3.3 mg/dL), increased transaminases and pleural effusion resulting in oxygen requirement (3/5 diagnostic pEBMT criteria). Reversal of portal venous flow was also observed. The patient was treated with defibrotide for 14 days, starting 1 day after RT, leading to prompt decrease in ascites and improvement in portal venous flow. In addition, bilirubin and transaminase levels decreased 1 and 4 days after initiating defibrotide, respectively, and coagulation improved. The patient was discharged after 16 days in good condition.

### 2.2 Patient 2 (Female, 12 Years)

Patient 2 received 6‐mercaptopurine and cytarabine within Consolidation A of AIEOP‐BFM ALL 2017. Two days after the last cytarabine dose was administered (Day 50 of Consolidation A), hyperbilirubinaemia (up to 5.7 mg/dL) was noted during outpatient monitoring and the patient was hospitalised, presenting with anaemia, coagulopathy, hepatomegaly, ascites and hepatic tenderness (3/5 diagnostic pEBMT criteria). Reduced hepatic perfusion was also observed. Defibrotide was administered for 11 days, starting 3 days after displaying hyperbilirubinaemia (1 day post‐hospitalisation for VOD/SOS symptoms), resulting in regression of hepatosplenomegaly and normalised hepatic perfusion post‐treatment. The patient was discharged after 13 days in good condition.

### 2.3 Patient 3 (Female, 6 Years)

Patient 3 received cyclophosphamide, thioguanine and cytarabine based on AIEOP‐BFM ALL 2017 protocol IIIB. Twenty‐six days after the second protocol IIIB was initiated, the patient presented with RT, anaemia and fever; other symptoms developing 2 days later were increased abdominal girth, hepatomegaly, increased transaminases, massive ascites requiring paracentesis and pleural effusion resulting in oxygen requirement (3/5 diagnostic pEBMT criteria). Reversal of portal venous flow and severely compromised renal function were also observed. The patient received defibrotide treatment for 14 days, starting 2 days after presentation of RT with rapid benefit. The patient was discharged after 17 days in good condition.

### 2.4 Patient 4 (Female, 11 Years)

Patient 4 had received daunorubicin, vincristine, pegylated asparaginase and prednisolone during protocol IA of AIEOP‐BFM ALL 2017. Four days after the last intravenous chemotherapy (vincristine and daunorubicin), during tapering of prednisone, the patient was hospitalised due to scleral icterus, hyperbilirubinaemia (up to 10.5 mg/dL) and hepatic tenderness. The patient also displayed anaemia, fever, thrombocytopaenia, coagulopathy, hepatomegaly with impaired perfusion and increased transaminases (3/5 diagnostic pEBMT criteria). Defibrotide was administered for 8 days, starting 1 day after the presentation of hyperbilirubinaemia. The patient was later transplanted and did not develop VOD/SOS in the post‐HSCT course. The patient was discharged after 11 days with resolution of symptoms.

### 2.5 Patient 5 (Female, 3 Years)

Patient 5 received thioguanine, cytarabine and cyclophosphamide based on AIEOP‐BFM ALL 2009 protocol IIB. One day after treatment ended, the patient was hospitalised due to fever and 2 days later displayed RT, anaemia, weight gain, increased abdominal girth, hepatomegaly and ascites (4/5 diagnostic pEBMT criteria). Reversal of portal venous flow was also observed. Defibrotide was administered for 18 days, starting at the presentation of weight gain. Post‐defibrotide treatment, ultrasound revealed a decreased liver size with improved perfusion and no ascites. The patient was discharged after 19 days in good condition.

### 2.6 Patient 6 (Male, 22 Months)

Patient 6 received cyclophosphamide, cytarabine and thioguanine based on AIEOP‐BFM ALL 2017 treatment protocol IIB. Twelve days after the start of protocol IIB, the patient presented with RT and increased transaminases followed by anaemia, coagulopathy, weight gain, hepatomegaly, ascites requiring paracentesis, hyperbilirubinaemia (up to 3.5 mg/dL) and bilateral pleural effusions (5/5 diagnostic pEBMT criteria). Reversal of portal venous flow was also observed. Defibrotide was administered for 17 days, starting 2 days after presentation of RT (1 day post‐hospitalisation for VOD/SOS symptoms), leading to gradual resolution of symptoms. The patient was discharged after 18 days in good condition.

With regard to further use of the potentially VOD/SOS‐associated medication, Patients 1, 3, 5 and 6 received 6‐mercaptopurine later in the protocol during maintenance, and no further VOD/SOS occurred. For Patient 2, 6‐mercaptopurine was restarted after recovery, and no further VOD/SOS occurred. Patient 4 was neither retreated with 6‐mercaptopurine nor thioguanine due to change of treatment indication (early HSCT indication).

## 3 Discussion

The presented case series demonstrate that severe VOD/SOS can develop following conventional chemotherapy and beyond a HSCT setting. Interestingly, these cases all occurred during first‐line treatment according to AIEOP‐BFM ALL protocol, mostly correlating with use of thiopurines (thioguanine or 6‐mercaptopurine). None of the patients (except possibly Patient 6 due to age) carried known pre‐existing high‐risk factors, such as infant age, iron overload, previous irradiation or high‐risk hepatic conditions [1, 2]. The presented symptoms of RT, coagulopathy, hepatomegaly (with hepatic tenderness), ascites, hyperbilirubinaemia and elevated transaminases did not differ from post‐HSCT VOD/SOS symptoms [2].

All patients were promptly diagnosed according to pEBMT criteria, with RT being the most sensitive trigger (4/6 patients), and treated with defibrotide within 1–2 days after being hospitalised with VOD/SOS symptoms; all also fulfilled Seattle and Baltimore diagnostic criteria but at later time points [16, 17]. Diagnosis according to pEBMT criteria was on average 2–4 days earlier than expected according to modified Seattle or Baltimore criteria [18, 19]. Although patients presented with severe grade 4 VOD/SOS as per pEBMT criteria [2], the treatment duration was less than the recommended ≥ 21 days since defibrotide was administered for 8–18 days only [11, 12]. Patients recovered quickly and were discharged after 11–19 days without fatalities. These successful outcomes mirrored other reports that most patients with ALL have recovered from VOD/SOS [7, 8].

Post‐HSCT VOD/SOS is a well‐recognised early transplant‐related complication [3] that needs early diagnosis and prompt intervention with defibrotide, both pivotal for positive outcomes [8, 15]. While prompt intervention with defibrotide might have affected VOD/SOS outcomes in these paediatric patients with ALL, VOD/SOS may have a more benign course in the non‐HSCT setting.

VOD/SOS in the non‐HSCT setting is a rare complication and, therefore, an often‐missed differential diagnosis, which could delay diagnosis [6]. In addition, VOD/SOS may be misdiagnosed as therapy‐related organ toxicity, progressing to multiorgan failure of unknown origin [2]. Therefore, awareness of non‐HSCT VOD/SOS needs to be raised amongst non‐HSCT healthcare professionals [2, 6, 8]. Due to overlapping clinical presentation of VOD/SOS in patients with conventional chemotherapy or post‐HSCT, pEBMT criteria can be applied in both scenarios to trigger prompt diagnosis and intervention [2, 6, 8]. Systematic analysis of prospective and retrospective occurrences of VOD/SOS in this setting may help identify unique treatment‐related risk factors, allowing implementation of a specific non‐HSCT VOD/SOS risk score.

Thioguanine is a well‐known risk factor for developing VOD/SOS [6, 7, 8]. The T‐IND study revealed that 31% of patients who developed VOD/SOS had received thioguanine [4]. In the cases reported here, 4/6 patients received thioguanine treatment and one patient received 6‐mercaptopurine as part of AIEOP‐BFM ALL 2009/2017 treatment protocol prior to developing VOD/SOS; thus, thiopurines may have contributed to VOD/SOS development in these patients. From the T‐IND study, it was also reported that 54%, 51% and 48% of patients who developed VOD/SOS had received cyclophosphamide, cytarabine and vincristine, respectively [4]. In the cases reported here, 3/6 patients received cyclophosphamide, 4/6 patients received cytarabine and one patient received vincristine as part of the AIEOP‐BFM ALL 2017 treatment protocol prior to developing VOD/SOS. This observation, alongside the data reported in the T‐IND study, suggests that these non‐transplant‐associated chemotherapy agents may have contributed to the development of VOD/SOS. In addition, it is noteworthy that Patient 4 received pegylated asparaginase, since a search of post‐marketing reports in the Food and Drug Administration (FDA) Adverse Event Reporting System database showed that long‐acting asparaginase products may have contributed to VOD/SOS development [20]. However, since only one patient developed VOD/SOS after pegylated asparaginase, no conclusions can be drawn. Differences in risk, incidence, timing and severity of VOD/SOS between an intensified multimodal chemotherapy protocol, such as AIEOP‐BFM ALL 2017, versus similar protocols are additional factors to be evaluated.

Although defibrotide was used beyond indication [21], VOD/SOS resolution in these non‐HSCT patients suggests similarity of the underlying pathophysiology with VOD/SOS post‐HSCT. Prior VOD/SOS is an established risk factor for recurrence of VOD/SOS. It is likely that prompt defibrotide treatment for chemotherapy‐induced VOD/SOS might have an impact on the incidence and morbidity of subsequent VOD/SOS post‐HSCT. Awareness and proper diagnosis of VOD/SOS will impact consecutive HSCT through appropriate conditioning regimens and earmarking high‐risk patients for pre‐emptive intervention or even defibrotide prophylaxis (which has demonstrated benefits in high‐risk paediatric patients) and is recommended by the FDA for prophylaxis of VOD/SOS in patients treated with lovotibeglogene autotemcel [13].

In conclusion, severe VOD/SOS occurred during first‐line treatment of ALL; prompt recovery was observed in these paediatric patients with non‐HSCT VOD/SOS following early diagnosis and initiation of defibrotide treatment.

## Author Contributions

Katharina Kleinschmidt, Selim Corbacioglu and Anja Troeger conceived the study and have written the manuscript. Jürgen Föll, Tarek Hanafee‐Alali, Marcus Jakob, Sonja Kramer and Silke Kietz included patients and provided the clinical data. All authors had directly accessed and verified the underlying data reported in the manuscript and approved the final manuscript.

## Ethics Statement

The authors have nothing to report.

## Consent

The parents of the paediatric patients provided written consent to publish this case series.

## Conflicts of Interest

Katharina Kleinschmidt received support for participation in an advisory board from Jazz Pharmaceuticals and congress sponsorship from Medac GmbH. Selim Corbacioglu received honorarium for advisory activity from Jazz Pharmaceuticals. The other authors declare no conflicts of interest.

## Data Availability

All data underlying the results are available as part of the article and no additional source data are required.
